# Supplementary material for: Content-rich biological network constructed by mining PubMed abstracts
Source: BMC Bioinformatics. 2004 Oct 8;5:147. doi: 10.1186/1471-2105-5-147 (PMC528731; doi:10.1186/1471-2105-5-147)
Supplement: Additional File 2 — The original results of the above study (non-essential files are deleted to keep the file size under the limit set by BMC bioinformatics). [file 1471-2105-5-147-S2.bz2 › chilibotAdditionalFile2/dip05/179ID9417082E617/html/PLAUR_LYN.html]

 


 **PLAUR** and **LYN** 
  
Found 2 abstracts in PubMed, retrieved 2.  
 

 What does Google say? 
 PDF only 
| .edu only 

---

- Int Immunol, 2002   **A novel lipid raft associated glycoprotein, TEC 21, activates rat basophilic leukemia cells independently of the type 1 Fc epsilon receptor.** .
  Recent data suggest that initiation of signal transduction via type 1 Fc epsilon receptor Fc epsilon RI and other immunoreceptors is spatially constrained to lipid rafts.
  In order to better understand the complexity and function of these structures, we prepared mAb against lipid rafts from the rat basophilic leukemia cell line, RBL 2H3, which is extensively used for analysis of Fc epsilon RI mediated activation.
  One of the antibodies was found to recognize a novel glycosylphosphatidylinositol anchored plasma membrane glycoprotein of 250 amino acids, designated TEC 21, containing a cysteine rich domain homologous to those found in the urokinase plasminogen activator receptor **[PLAUR]** Ly 6 snake neurotoxin family.
  TEC 21 is abundant on the surface of RBL 2H3 cells >10 6 molecules cell , but is absent in numerous rat tissues except for testes.
  Aggregation of TEC 21 on RBL 2H3 cells induced a rapid increase in tyrosine phosphorylation of several substrates including Syk kinase and LAT adaptor, calcium flux, and release of secretory components.
  Similar but more profound activation events were observed in cells activated via Fc epsilon RI.
  However, aggregation of TEC 21 did not induce changes in density of IgE Fc epsilon RI complexes, tyrosine phosphorylation of Fc epsilon RI beta and gamma subunits, and co aggregation of **Lyn** kinase.
  TEC 21 induced activation events were also observed in Fc epsilon RI mutants of RBL 2H3 cells.
  Thus, TEC 21 is a novel lipid raft component of RBL 2H3 cells whose aggregation induces activation independently of Fc epsilon RI.
